# A comparison of bioelectrical impedance analysis and air displacement plethysmography to assess body composition in children

**DOI:** 10.3389/fpubh.2023.1164556

**Published:** 2023-07-04

**Authors:** Fangfang Chen, Lijun Wu, Yiren Chen, Jing Wang, Junting Liu, Guimin Huang, Dongqing Hou, Zijun Liao, Ting Zhang, Xianghui Xie, Gongshu Liu

**Affiliations:** ^1^Department of Epidemiology, Capital Institute of Pediatrics, Beijing, China; ^2^Tianjin Women's and Children's Health Center, Tianjin, China

**Keywords:** bioelectrical impedance analysis, air displacement plethysmography, body composition, obesity, children

## Abstract

**Background:**

Accurate assessment of body composition (BC) is important to investigate the development of childhood obesity. A bioelectrical impedance analysis (BIA) device is portable and inexpensive compared with air displacement plethysmography (ADP) for the assessment of BC and is widely used in children. However, studies of the effectiveness of BIA are few and present different results, especially in pediatric populations. The aim of this study was to evaluate the agreement between BIA and ADP for estimating BC.

**Methods:**

The BC of 981 Chinese children (3–5 years) was measured using the BIA device (SeeHigher BAS-H, China) and ADP (BOD POD).

**Results:**

Our results showed that BIA underestimated fat mass (FM) and overestimated fat-free mass (FFM) in normal weight children (*P* < 0.05), but the opposite trend was shown in children with obesity (*P* < 0.05). The agreement between FM and FFM measured by the two methods was strong (CCC > 0.80). The linear regression equation of 5-year-old children was constructed.

**Conclusion:**

The SeeHigher BAS-H multi-frequency BIA device is a valid device to evaluate BC in Chinese preschool children compared with ADP (BOD POD), especially in 5-year-old children or children with obesity. Further research is needed to standardize the assessment of BC in children.

## Introduction

Obesity and overweight are defined as an abnormally high or excessive accumulation of fat according to the World Health Organization. Childhood obesity is a growing public health concern worldwide (1), and the prevalence of childhood obesity has significantly increased in China (2, 3). Children with overweight and obesity are more likely to become adults with obesity, and childhood obesity strongly predisposes them to some adult diseases (4). Obesity is associated with an increased risk of a wide array of health problems, including hypertension, type 2 diabetes, and cardiovascular disease (5).

Body mass index (BMI), defined as the body mass in kilograms divided by the height in meters squared, is a widely used measure of overweight and obesity. It is considered easy to apply; however, BMI is limited by not distinguishing between different compartments of body composition. In other words, BMI provides no information about fat mass (FM) and fat-free mass (FFM), which confer different health outcomes. Studies have indicated that the distribution and location of adiposity are associated with an increased risk of metabolic diseases (6, 7). Estimating FM has been shown to be a better predictor of adiposity-related metabolic risk than BMI due to the negative consequences of fat accumulation (8, 9). Therefore, the evaluation of body composition has been recommended as a complementary assessment for the diagnosis of obesity.

At present, several techniques are utilized for body composition analysis (BCA), such as anthropometric estimates, dual-energy X-ray absorptiometry (DXA), magnetic resonance imaging (MRI), computed tomography (CT), air displacement plethysmography (ADP), and bioelectrical impedance analysis (BIA) (10). Among these techniques, DXA and CT use radiation, which limits the number of repeated measures possible. Although anthropometric measures are simple, inexpensive, and easy to use, the best anthropometric measures to assess risks related to adiposity have not been established. Of the currently available body composition techniques, ADP and BIA have become widely used BCA methods in research and clinical settings (11–14). Both methods could derive precise BCA results, but ADP and BIA are different and theory-based and have their own advantages.

ADP uses air displacement within a two-chambered air-filled closed system as an alternative to water displacement and measures full body densitometry (15). BOD POD^®^ is a commercially available ADP instrument for assessing body composition that is used in clinical, commercial, and research settings (16). ADP offers a fast, accurate, and reliable means of assessing body composition in children (17).

BIA is used to assess body composition based on the electrically conductive properties of the body through painless electrical currents throughout the body to measure impedance (18). There are single-frequency BIA, multi-frequency BIA (MFBIA), and bioelectrical impedance spectroscopy. Following advances in BIA technology, BIA equipment has been developed and has become the most common and affordable device for assessing body composition (19).

In contrast to ADP, BIA is a relatively inexpensive and portable method for assessing body composition. However, the validity of the technique compared with ADP presents different results, and very little research has been conducted in the preschool pediatric population. Comparative studies regarding these two methods among Chinese children are greatly needed. The purpose of the present study was to compare the two widely used BCA methods, MFBIA and ADP, among Chinese preschool children.

## Methods

### Study design and participants

The study compared two body composition measurement tools: (a) MFBIA and (b) ADP (BOD POD). The ADP was used as a standard against which the MF BIA was compared. In China, children generally enter kindergarten at ~3 years old and stay there for 3 years (i.e., junior, middle, and senior classes) before elementary school, so most kindergarten children are between 3 and 6 years old. We recruited 1,011 Chinese children from three kindergartens in the Jinghai District in Tianjin between September 2020 and December 2020. Under the care of their parents and the guidance of professional researchers, 1,011 children underwent body composition measurements using ADP, but 30 of them were excluded due to missing BIA measurements. We obtained written informed consent from all children's guardians. The studies involving human participants were reviewed and approved by the IRB of Tianjin Women's and Children's Health Center (BGI-IRB 17116-201711). This study followed the Strengthening the Reporting of Observational Studies in Epidemiology (STROBE) reporting guidelines for cross-sectional studies.

### Study protocol

To address potential sources of bias, all assessments were conducted by trained data collectors, most of whom were nurses, and school doctors. A set of strategies was implemented for data quality control. First, quality control of examinations was performed by the same professional researchers, who strictly followed a standardized protocol. Second, all participants fasted after 20:00 the day before the physical examination, fasted on the day of the examination, and then underwent BIA and ADP (BOD POD) to measure FM and FFM on the same morning. The height of the children was measured by trained staff using wall-mounted stadiometers, and the weight was measured by ADP, both accurate to 0.1 cm. Then, BMI was calculated. The z scores for weight-for-height and BMI-for-age were calculated using ADP measurements, and according to the WHO standard, overweight or obesity in children was defined by weight-for-height or BMI-for-age of 2 and 3 for children under 5 years old and BMI-for-age of 1 and 2 for children above 5 years old (20). Furthermore, all instruments used were the same in the three kindergartens during the survey.

### Multi-frequency bioelectrical impedance analysis

MFBIA measurements were conducted using BIA (SeeHigher BAS-H, China), which measured impedance at varying frequencies (1, 5, 50, 250, 500, and 1,000 kHz) across the legs, arms, and trunk. Children were required to be fasting and have an empty bladder. When in the measurement, children in light clothing stood on the platform without shoes and held both hands at a 45-degree angle away from the body; four tactile electrodes were in contact with the palm and thumb of both hands, and the other four were in contact with the anterior and posterior aspects of the sole of both feet. The measurements were collected, and then, the FM and FFM were calculated by an undisclosed proprietary algorithm.

### Air displacement plethysmography

FM and FFM were assessed by ADP using the pediatric option of the BOD POD Gold Standard Body Composition Tracking System (COSMED USA, Inc., Concord, CA). Body mass was measured using an electronic scale, and body volume was assessed in a closed chamber utilizing the relationship between pressure and volume. Children entered the ADP system without shoes in a tightfitting swimsuit and a swimming cap, and their total body volume was measured. Volume measurements were always performed in triplicate and strictly according to the manufacturer's instructions. Body volume was corrected for surface area artifacts and thoracic gas volume. Surface area artifacts and thoracic gas volume were estimated based on the equations that were developed and built into the machine.

### Statistical analysis

Descriptive characteristics of the participants, FM, and FFM were described as mean ± standard deviation (SD). The independent *t*-test was used to compare the differences in the anthropometric measures of the participants between boys and girls, and a paired *t*-test was used to compare the differences between FM and FFM measured by BIA and ADP. The root mean square error (RMSE) is derived from the linear regression results of FM or FFM measured using the BIA device or ADP and used to represent the absolute value of the difference between the measurements of the two methods. Lin's concordance correlation coefficient (CCC) and Bland–Altman analysis were used to evaluate the agreement between the two methods. To eliminate proportion bias, the data were logarithmically transformed, and then, the Bland–Altman analysis was performed (21). Controlling for covariates (including age, sex, and BMI), the split-group approach in cross-validation was used to fit the linear regression prediction equation with BIA measurements as independent variables and ADP measurements as dependent variables. All statistical analyses were performed using SPSS 26.0, R software 4.1.2, and Mplus 8.0, and a bilateral *P-*value of < 0.05 was considered to be statistically significant.

## Results

After excluding 30 children due to missing BIA measurements, 981 children with both ADP and BIA measurements were enrolled in our study (513 boys and 468 girls, aged 3 to 5 years). An independent *t*-test analysis of the enrolled and excluded children showed that there was no significant difference in sex, age, BMI, and FFM level by ADP between these two groups, but there was a significant difference in FM measurement (*P* = 0.047). The descriptive characteristics of the study sample reported in Table 1 include the anthropometric measures of the participants and their mean FM and FFM values as determined using BIA and ADP. Significant differences were observed between boys and girls for height, weight measured by ADP, and FFM measured by BIA or ADP. There were no statistically significant differences between boys and girls in terms of age, BMI, and FM measured by BIA or ADP.

**Table 1:** Descriptive characteristics of the study sample.

	**All (*N* = 981)**	**Boys (*n* = 513)**	**Girls (*n* = 468)**	P^*^
Age (yrs)	4.50 ± 0.84	4.52 ± 0.84	4.48 ± 0.83	0.390
BMI by ADP (kg/m^2^)	15.6 ± 1.9	15.7 ± 1.9	15.5 ± 1.8	0.109
Height (cm)	107.8 ± 7.3	108.5 ± 7.6	107.1 ± 6.8	**0.002**
Weight by ADP (kg)	18.4 ± 4.0	18.7 ± 4.3	18.0 ± 3.7	**0.002**
FM by ADP (kg)	4.0 ± 1.9	4.1 ± 2.0	3.9 ± 1.8	0.127
FM by BIA (kg)	3.4 ± 2.1	3.4 ± 2.2	3.4 ± 2.0	0.873
FFM by ADP (kg)	14.4 ± 3.0	14.7 ± 3.2	14.1 ± 2.7	**0.002**
FFM by BIA (kg)	15.1 ± 2.5	15.6 ± 2.6	14.6 ± 2.1	**< 0.001**

^*^P-values are from tests compared between boys and girls by independent t-tests; bold means bilateral P < 0.05. ADP, air displacement plethysmography; BIA, bioelectrical impedance analysis; BMI, body mass index; FM, fat mass; FFM, fat-free mass. Those highlighted in bold indicate that the associations showed statistical significance.

Table 2 shows the difference and comparison between FM and FFM measured by BIA and ADP stratified by sex. In all boys and girls, the mean FM measured by BIA was lower than that measured by ADP (*P* < 0.001), and the RMSE was 1.178 and 1.068 kg, respectively. Stratified by age, the mean FM measured by BIA of the 3- and 4-year-old groups was also lower than that measured by ADP (*P* < 0.05), and the RMSE of all groups was within 1.6 kg. Stratified by BMI, compared with ADP measurements, both boys and girls with normal weight had lower FM and higher FFM measured by BIA (*P* < 0.05). In contrast, both boys and girls with obesity had higher FM and lower FFM (*P* < 0.05), and the RMSE was 1.341 and 1.235 kg, respectively. Stratified by age, the mean FFM measured by BIA of the 3- and 4-year-old groups was also higher than that measured by ADP (*P* < 0.05), and the RMSE of all groups was within 1.7 kg. Stratified by BMI, the mean FFM measured by BIA was higher than that measured by ADP in both boys and girls of normal weight (*P* < 0.05).

**Table 2:** Difference between BIA and ADP measurements of FM and FFM in children aged 3 to 5 years.

	**Boys**					**Girls**				
	***n***	**BIA**	**ADP**	***P* ^a^**	**RMSE**	***n***	**BIA**	**ADP**	***P* ^a^**	**RMSE**
**FM (kg)**										
All	513	3.4 ± 2.2	4.1 ± 2.0	**< 0.001**	1.178	468	3.4 ± 2.0	3.9 ± 1.8	**< 0.001**	1.068
**Age (yrs)**										
3	163	2.7 ± 1.2	3.5 ± 1.4	**< 0.001**	1.075	161	2.9 ± 1.3	3.3 ± 1.4	**< 0.001**	1.008
4	200	3.5 ± 2.2	4.6 ± 1.8	**< 0.001**	1.086	175	3.4 ± 2.0	4.3 ± 1.7	**< 0.001**	0.986
5	150	3.9 ± 2.8	4.0 ± 2.6	0.249	1.213	132	4.0 ± 2.4	4.0 ± 2.3	0.725	0.987
**BMI groups** ^b^										
Normal	449	2.7 ± 1.0	3.6 ± 1.3	**< 0.001**	1.094	421	2.9 ± 1.2	3.5 ± 1.3	**< 0.001**	1.069
Overweight	29	5.8 ± 1.4	5.8 ± 2.0	0.958	1.527	30	6.5 ± 1.7	6.4 ± 1.6	0.568	1.071
Obesity	35	9.5 ± 3.0	8.5 ± 3.4	**< 0.001**	1.429	17	9.5 ± 2.3	8.9 ± 2.3	**0.014**	0.979
**FFM (kg)**										
All	513	15.6 ± 2.6	14.7 ± 3.2	**< 0.001**	1.341	468	14.6 ± 2.1	14.1 ± 2.7	**< 0.001**	1.235
**Age (yrs)**										
3	163	13.4 ± 1.4	12.3 ± 1.6	**< 0.001**	1.103	161	13.0 ± 1.2	12.4 ± 1.4	**< 0.001**	0.967
4	200	15.5 ± 1.9	14.1 ± 2.3	**< 0.001**	1.259	175	14.6 ± 1.5	13.4 ± 1.8	**< 0.001**	1.151
5	150	18.0 ± 2.3	18.0 ± 2.8	0.752	1.360	132	16.7 ± 1.9	17.0 ± 2.5	0.053	1.129
**BMI groups** ^b^										
Normal	449	15.1 ± 2.2	14.0 ± 2.6	**< 0.001**	1.225	421	14.3 ± 1.8	13.6 ± 2.2	**< 0.001**	1.203
Overweight	29	17.6 ± 2.4	17.6 ± 2.7	0.995	1.637	30	17.2 ± 1.9	17.5 ± 2.6	0.194	1.448
Obesity	35	20.0 ± 2.5	20.8 ± 2.9	**0.001**	1.424	17	19.3 ± 1.8	20.2 ± 2.3	**0.002**	0.964

^a^The P-value is from the paired t-tests. ^b^BMI groups are determined by the WHO 2006 standard based on ADP measurements (20). ADP, air displacement plethysmography; BIA, bioelectrical impedance analysis; BMI, body mass index; FM, fat mass; FFM, fat-free mass; RMSE, root mean square error. Those highlighted in bold indicate that the associations showed statistical significance.

Table 3 shows the agreement between BIA and ADP in the measurement of FM and FFM. In general, with increasing age and BMI for boys and girls, the agreement between BIA and ADP gradually increased, especially in 5-year-old boys and girls and obese boys and girls, and the consistency was better (CCC > 0.80).

**Table 3:** Consistency analysis of BIA and ADP measurements of FM and FFM in children aged 3–5 years.

	**Boys**				**Girls**			
	***n***	**CCC^a^**	**Log (BIA) - log (ADP)**	**LoA**	***n***	**CCC^a^**	**Log (BIA)-log (ADP)**	**LoA**
**FM (kg)**								
All	513	0.76	−0.105	−0.470 to 0.261	468	0.79	−0.067	−0.408 to 0.274
**Age (yrs)**								
3	163	0.50	−0.119	−0.476 to 0.238	161	0.67	−0.053	−0.396 to 0.290
4	200	0.70	−0.147	−0.484 to 0.189	175	0.70	−0.131	−0.452 to 0.191
5	150	0.88	−0.031	−0.402 to 0.340	132	0.90	−0.001	−0.308 to 0.307
**BMI groups** ^b^								
Normal	449	0.36	−0.125	−0.488 to 0.237	421	0.54	−0.076	−0.427 to 0.274
Overweight	29	0.62	0.020	−0.281 to 0.321	30	0.74	0.006	−0.187 to 0.199
Obesity	35	0.86	0.058	−0.100 to 0.217	17	0.88	0.034	−0.079 to 0.147
**FFM (kg)**								
All	513	0.85	0.029	−0.052 to 0.110	468	0.85	−0.019	−0.055 to 0.093
**Age (yrs)**								
3	163	0.58	0.038	−0.036 to 0.112	161	0.64	0.018	−0.047 to 0.084
4	200	0.68	0.041	−0.039 to 0.122	175	0.61	0.038	−0.031 to 0.108
5	150	0.86	0.003	−0.061 to 0.067	132	0.85	−0.006	−0.064 to 0.052
**BMI groups** ^b^								
Normal	449	0.79	0.034	−0.042 to 0.111	421	0.78	0.022	−0.050 to 0.095
Overweight	29	0.79	0.000	−0.080 to 0.082	30	0.79	−0.007	−0.073 to 0.060
Obesity	35	0.83	−0.017	−0.073 to 0.040	17	0.81	−0.019	−0.056 to 0.019

^a^All P < 0.001. ^b^BMI groups are determined by the WHO 2006 standard based on ADP measurements (20). ADP, air displacement plethysmography; BIA, bioelectrical impedance analysis; BMI, body mass index; FM, fat mass; FFM, fat-free mass; CCC, Lin's concordance correlation coefficient; LoA, limit of agreement.

**Figure 1:**
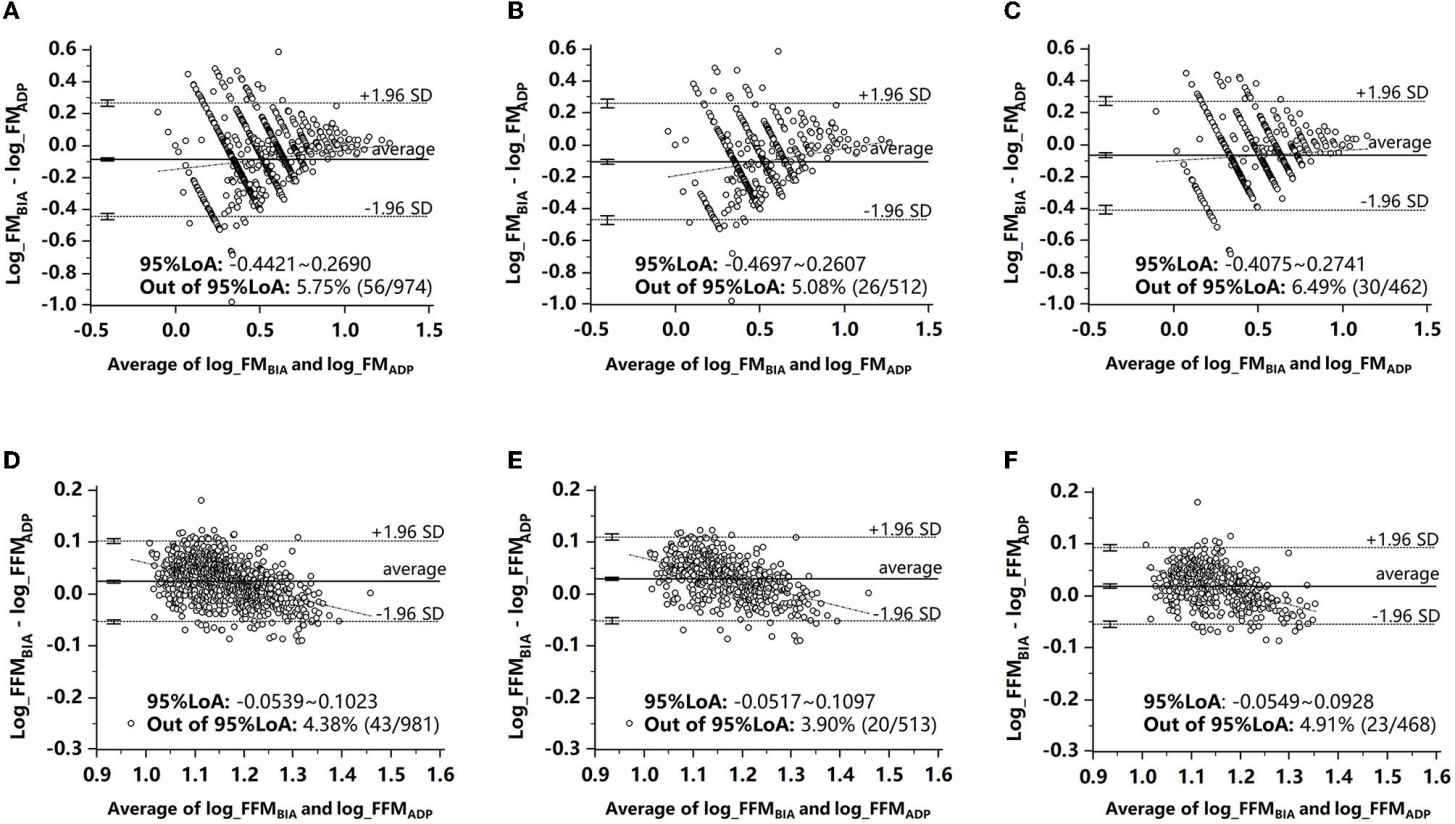
Bland–Altman analysis plots of agreement between FM and FFM measured by BIA and ADP. **(A)** FM in all children. **(B)** FM in boys. **(C)** FM in girls. **(D)** FFM in all children. **(E)** FFM in boys. **(F)** FFM in girls. ADP, air displacement plethysmography; BIA, bioelectrical impedance analysis; FM, fat mass; FFM, fat-free mass.

The Bland–Altman analysis plots of agreement between FM and FFM measured by BIA and ADP are shown in Figure 1. To eliminate proportional bias, the Bland–Altman analysis was performed after the logarithmic conversion of data. As shown in Figure 1, the LoA range of FM was wider than that of FFM. Supplementary Figure 1 shows the Bland–Altman plot stratified by age. FM measured by BIA and ADP had good consistency only in 5-year-old children, and the percentage of points beyond the 95% agreement limit was < 5% (14/282, 4.96%). FFM measurements in 4- and 5-year-old children had good consistency, and the proportion of points beyond the 95% agreement limit was < 5% (11/375, 2.93%; 13/282, 4.61%). Supplementary Figure 2 shows the Bland–Altman plot stratified by BMI. FM and FFM measured by BIA and ADP had good consistency in children with obesity, and the proportion of points beyond the 95% agreement limit was < 5% (2/52, 3.85%). In conclusion, the LoA ranges of FM and FFM narrowed with age or BMI in both boys and girls, suggesting that in children with older age or larger body weight, the agreement of body composition measured by ADP and BIA was higher.

Table 4 shows the classification of FM measured by BIA or ADP in children aged 3 to 5 years. The FM and FFM measured by BIA and ADP were divided into three categories (Q1/Q2/Q3) by sex and age. With ADP classification as the standard, the correct classification rates of FM and FFM in boys were 56.92 and 65.30%, respectively, and those in girls were 62.82 and 64.32%, respectively. In addition to the poor agreement of FM classification in boys (kappa value was 0.350), the agreement of FFM classification in boys and the agreement of FM and FFM classification in girls were all good, and kappa values were 0.480, 0.442, and 0.465, respectively (*P* < 0.001). Due to the limitation of sample size, overweight and obesity were combined into the “non-normal weight group,” and the agreement of FM classification in all groups was poor (kappa values < 0.40). However, in girls, the agreement of FFM classification in all weight groups was good (kappa value > 0.40). In boys, the agreement of FFM classification was good (kappa value was 0.446) in the normal weight group but poor in the non-normal weight group (kappa value was 0.355, *P* < 0.001).

**Table 4:** Classification and comparison of different methods to measure FM and FFM in children aged 3 to 5 years.

**Sex**	**FM**_**BIA**_			***n***	**FM_ADP_**	**FFM_BIA_**	***n***	**FFM**_**ADP**_		
			**Q1**	**Q2**	**Q3**			**Q1**	**Q2**	**Q3**
Boys	Q1	118	73 (44.51)	43 (22.63)	2 (1.26)	Q1	174	122 (69.71)	41 (25.15)	11 (6.29)
	Q2	213	75 (45.73)	100 (52.63)	38 (23.90)	Q2	175	48 (27.43)	88 (53.99)	39 (22.29)
	Q3	182	16 (9.76)	47 (24.74)	119 (74.84)	Q3	164	5 (2.86)	34 (20.86)	125 (71.43)
Girls	Q1	169	114 (69.94)	44 (32.12)	11 (6.55)	Q1	155	107 (70.39)	38 (23.17)	10 (6.58)
	Q2	154	35 (21.47)	71 (51.82)	48 (28.57)	Q2	150	40 (26.32)	81 (49.39)	29 (19.08)
	Q3	145	14 (8.59)	22 (16.06)	109 (64.88)	Q3	163	5 (3.29)	45 (27.44)	113 (74.34)

ADP, air displacement plethysmography; BIA, bioelectrical impedance analysis; FM, fat mass; FFM, fat-free mass.

Table 5 shows the linear regression association of FM and FFM measured by BIA and ADP in children of different ages. With FM or FFM measured by ADP as the dependent variable and FM or FFM measured by BIA as the independent variable, sex (one boy and two girls), age, and BMI were adjusted. The *R*^2^ of the linear regression association increased with age. This suggests that the linear regression association between ADP and BIA measurements was stronger in the 5-year-old children. Thus, the linear regression equation of 5-year-old children was conducted as follows:

FM: FM_ADP_ = 0.660^*^FM_BIA_ - 0.211^*^sex - 1.403^*^age + 0.173^*^BMI, *R*^2^ = 0.855;

FFM: FFM_ADP_ = 0.890^*^FFM_BIA_ + 0.216^*^sex + 2.010^*^age + 0.267^*^BMI, *R*^2^ = 0.850.

**Table 5:** Linear regression association of FM and FFM measured by BIA and ADP in children aged 3–5 years.

	**FM_BIA_**	**Sex**	**Age**	**BMI**	***R* ^2^**	***RMSE***
**FM**_ADP_~**FM**_BIA_						
3-year-old	0.547	−0.242	−0.019	0.193	0.475	1.109
4-year-old	0.389	−0.006	0.188	0.315	0.708	1.065
5-year-old	0.660	−0.211	−1.403	0.173	0.855	1.097
**FFM**_ADP_~**FFM**_BIA_						
3-year-old	0.605	0.580	0.873	0.194	0.612	1.059
4-year-old	0.713	0.132	0.513	0.342	0.798	0.933
5-year-old	0.890	0.216	2.010	0.267	0.850	1.115

Sex (one boy and two girls), age, and BMI were adjusted. BIA, bioelectrical impedance analysis; BMI, body mass index; FM, fat mass; FFM, fat-free mass. RMSE, root mean square error.

## Discussion

There are various techniques available for body composition assessment. The researcher should make an informed decision and choose the most appropriate method for BCA regarding accuracy, availability, validity, safety, and cost. In the present study, we compared the validity, differences, and precision between BIA and ADP in Chinese children from 3 to 5 years of age. Compared with ADP as a standard, our results showed that BIA underestimated FM and overestimated FFM in normal weight children (*P* < 0.05), but the opposite trend was shown in children with obesity (*P* < 0.05). The agreement between FM and FFM measured by the two methods was strong (CCC > 0.80), and with increasing body size (including age or BMI), the correlation was gradually enhanced. Thus, the linear regression equation of 5-year-old children was constructed.

In previous studies, the validity of BIA compared with ADP presented inconsistent results. Perteet-Jackson et al. (22) demonstrated that percent body fat (BF), FM, and FFM measures determined by MFBIA and ADP were not significantly different in American children. Sullivan et al. (23) also reported strong relationships between MFBIA and ADP measures of percent BF, FM, or FFM in an adult population. Vicente-Rodríguez et al. (24) demonstrated that ADP showed close agreement with BIA in European adolescents. However, compared with ADP, estimates of FFM from bioelectrical impedance spectroscopy mixture theory prediction were inaccurate among a large multi-ethnic cohort of infants from the United Kingdom, Singapore, and New Zealand (25).

More studies have shown that, for these two methods, there is a need for validity investigations, and they should not be used interchangeably. Fahs et al. (26) demonstrated that BIA used in BCA underestimated the percent BF compared with the ADP method. Nickerson et al. (27) examined the agreement between BIA, ADP, and DXA for estimating body composition in adults with obesity. The results found that BIA devices revealed proportional bias for percent BF and FFM when compared to ADP and DXA. This suggests that BIA is not acceptable for individual estimates of body composition in adults with obesity. Ferri-Morales et al. (28) reported that BIA underestimates percent BF compared with ADP in adolescents of southwest England. A previous study showed that good agreement and interchangeability of these two methods were not found in 7- to 13-year-old Belgian boys (29). Mahaffey et al. (30) reported that BIA and ADP cannot be used interchangeably in boys aged 6 to 12 years in the UK. Compared with the percent BF by ADP, the percent BF by BIA was significantly underestimated in this cohort. A systematic review showed that the validity of estimating body composition by BIA compared with ADP was inferior in children (31).

As technology advances, the accuracy and precision of BIA devices continue to improve, and compared with ADP, BIA has a more prominent portability advantage in follow-up studies. Whether BIA can be used as a substitute for ADP to measure body composition in the clinic and research remains to be determined.

This is the first study on the agreement between BIA and ADP for the measurement of body composition (FM and FFM) in Chinese children aged 3–5 years. A variety of indicators were used to evaluate the results of the two methods, and linear regression equations of the BIA and ADP measurements in 5-year-old children were also developed. In an environment of increasing concern for children's health and obesity development, our results provide strong evidence for the feasibility of BIA as a substitute for ADP. As a relatively inexpensive, convenient, and reliable method of body composition measurement, BIA is expected to be widely used in large-scale pediatric population screening or clinical investigation in the future.

However, this study is not without limitations. First, Bland–Altman analysis after logarithmic transformation would reduce the ratio bias but could not completely eliminate it, which might lead to a wide range of estimated LoA. The proportion bias may be caused by various reasons: Preschool children are at the peak stage of growth and development, and there are significant individual differences in the proportion of body parts with age and sex, which may affect the calculation results of BIA. In addition, the high water content of children's bodies can also cause errors in BIA calculations. To avoid scale bias, it has been found that BIS combined with the body geometry correction factor *K*_*B*_ (calculated from body measurements) (32) can account for different body geometries between individuals and better distinguish the relative differences in shape and size of body parts (legs, torso, and arms), thus accurately predicting body composition. However, there are still few data on children with *K*_*B*_, and more child measurements are needed to calibrate the *K*_*B*_. Second, although the two methods showed better consistency in measuring body composition in children with obesity than in normal or overweight children, due to the small number of children with obesity in this study, it is necessary to verify the results in prospective investigations with larger sample sizes.

In this study, the consistency and the rate of correct classification of FM were lower than those of FFM. The reason might be that ADP evaluates FM and FFM based on body densitometry at the same time, while BIA evaluates FFM first and then uses the total weight to subtract FFM to calculate FM. This results in a higher measurement error for FM than for FFM.

When comparing the differences, it was found that the difference between FM and FFM was statistically significant only in children of normal weight. In addition, the CCC values for evaluating consistency increased with age or BMI, and the LoA range in combined Bland–Altman analysis also showed a decreasing trend with increasing age or BMI. This might be because the BIA calculation formula is mostly based on adult data, and body water content was negatively correlated with age and BMI. As the body composition of children becomes more similar to that of adults as their age or BMI increases, the difference between the two methods decreases.

In addition, our results showed that BIA underestimated FM and overestimated FFM in normal weight children (*P* < 0.05), but the opposite trend was shown in children with obesity (*P* < 0.05). The effects of obesity status on the measurement of body composition by BIA can cause an increase in relative extracellular water in children and affect the hydration status, resulting in the overestimation of FM measured by BIA (33), but the difference between BIA and ADP was not statistically significant.

## Conclusion

Our findings indicate that the SeeHigher BAS-H MFBIA device produces similar body composition values as ADP. The agreement between FM and FFM measured by the two methods was strong, and with increasing body size (including age and BMI), the consistency between BIA and ADP was gradually enhanced. MFBIA (SeeHigher BAS-H, China) could provide a portable alternative to ADP in clinical and research settings for the assessment of body composition in older or higher BMI children. Further research is needed to standardize the assessment of body composition in children.

## Data availability statement

The original contributions presented in the study are included in the article/Supplementary material, further inquiries can be directed to the corresponding authors.

## Ethics statement

The studies involving human participants were reviewed and approved by the IRB of Tianjin Women's and Children's Health Center (BGI-IRB 17116-201711). Written informed consent to participate in this study was provided by the participants' legal guardian/next of kin.

## Author contributions

FC conceptualized and designed the study, carried out the analyses, and reviewed and revised the manuscript. GL conceptualized the study, supervised data analyses, and reviewed the manuscript. LW and YC analyzed the data and wrote the initial draft of the manuscript. JW, JL, GH, DH, and ZL were involved in data acquisition and data processing. XX and TZ conceptualized and supervised data analyses. All authors critically reviewed the manuscript for interpretation and intellectual content and approved the final manuscript as submitted.

## Supplementary material

Supplementary Figure 1 Bland–Altman analysis plots of agreement between FM and FFM stratification by age measured by BIA and ADP. **(A)** FM in 3-year-olds. **(B)** FM in 4-year-olds. **(C)** FM in 5-year-olds. **(D)** FFM in 3-year-olds. **(E)** FFM in 4-year-olds. **(F)** FFM in 5-year-olds. ADP, air displacement plethysmography; BIA, bioelectrical impedance analysis; FM, fat mass; FFM, fat-free mass.

Supplementary Figure 2 Bland–Altman analysis plots of agreement between FM and FFM stratification by BMI measured by BIA and ADP. **(A)** FM in normal children. **(B)** FM in children with overweight. **(C)** FM in children with obesity. **(D)** FFM in normal children. **(E)** FFM in children with overweight. **(F)** FFM in children with obesity. ADP, air displacement plethysmography; BIA, bioelectrical impedance analysis; FM, fat mass; FFM, fat-free mass.
