# Supplementary figures and images for: A comparison of bioelectrical impedance analysis and air displacement plethysmography to assess body composition in children
Source: Front Public Health. 2023 Jul 4;11:1164556. doi: 10.3389/fpubh.2023.1164556 (PMC10352489; doi:10.3389/fpubh.2023.1164556)

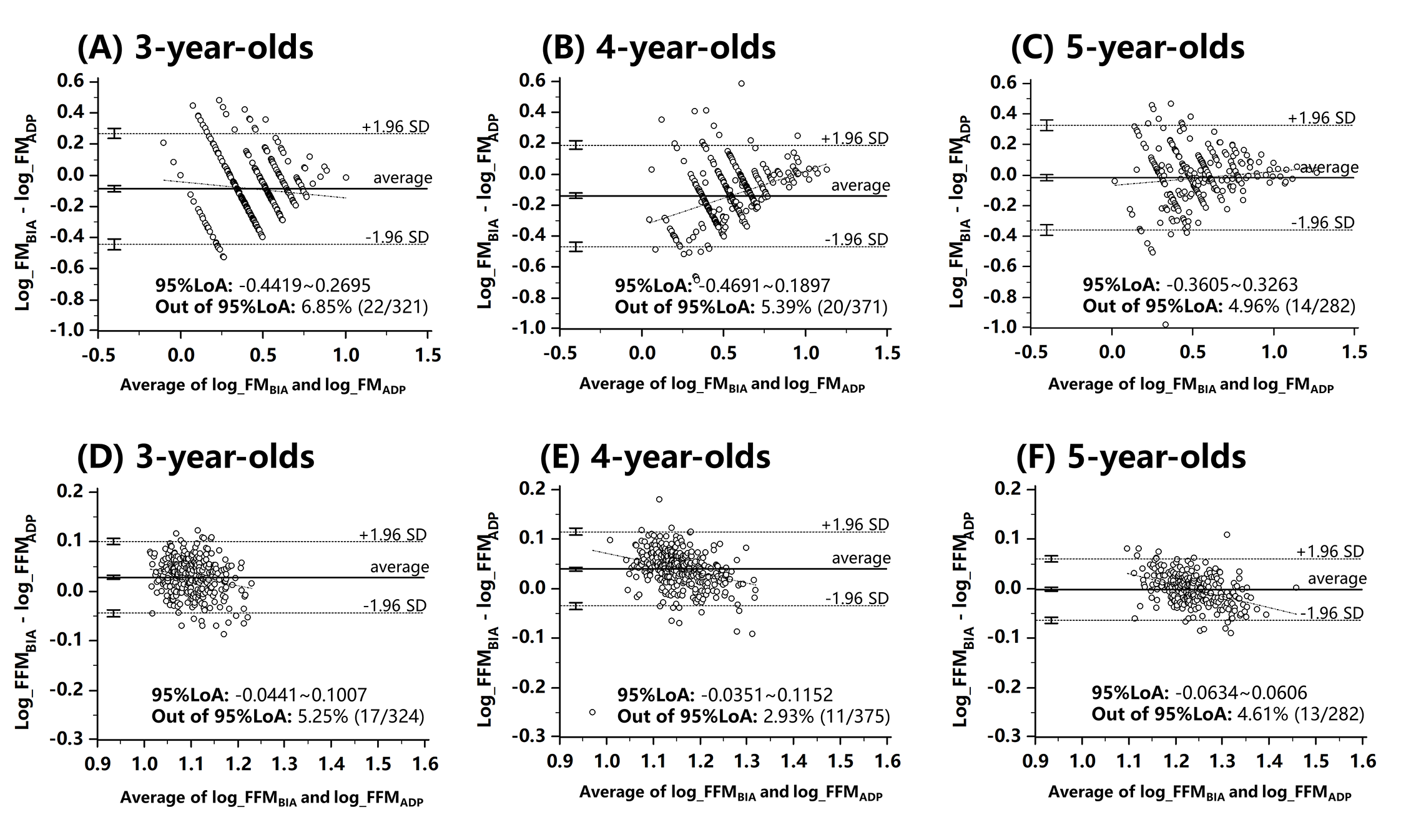

Supplement: Supplementary Figure 1 — Bland–Altman analysis plots of agreement between FM and FFM stratification by age measured by BIA and ADP. (A) FM in 3-year-olds. (B) FM in 4-year-olds. (C) FM in 5-year-olds. (D) FFM in 3-year-olds. (E) FFM in 4-year-olds. (F) FFM in 5-year-olds. ADP, air displacement plethysmography; BIA, bioelectrical impedance analysis; FM, fat mass; FFM, fat-free mass. [file Image_1.TIF]

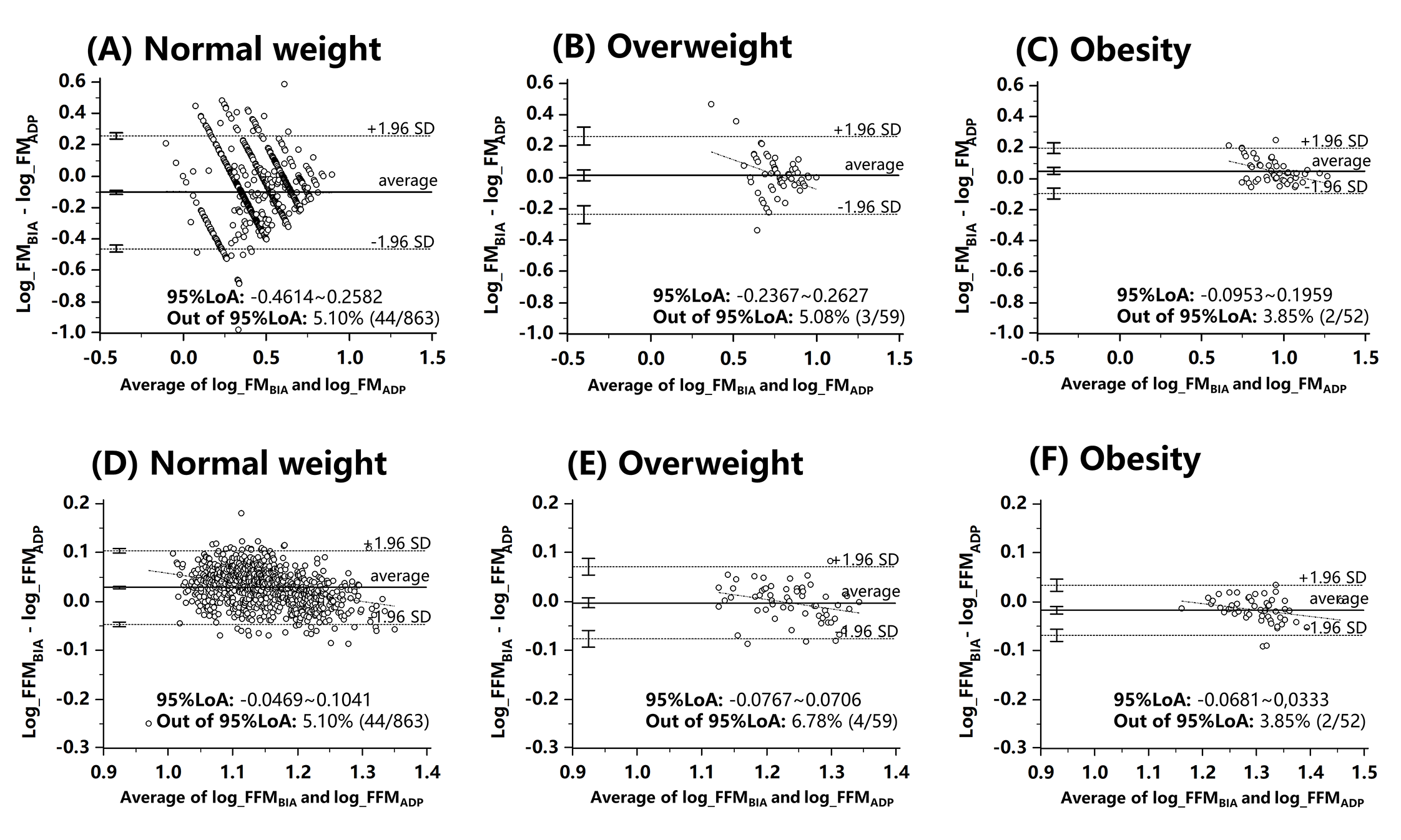

Supplement: Supplementary Figure 2 — Bland–Altman analysis plots of agreement between FM and FFM stratification by BMI measured by BIA and ADP. (A) FM in normal children. (B) FM in children with overweight. (C) FM in children with obesity. (D) FFM in normal children. (E) FFM in children with overweight. (F) FFM in children with obesity. ADP, air displacement plethysmography; BIA, bioelectrical impedance analysis; FM, fat mass; FFM, fat-free mass. [file Image_2.TIF]
